# Event versus activity-based cues and motivation in school-related prospective memory tasks

**DOI:** 10.1371/journal.pone.0215845

**Published:** 2019-04-19

**Authors:** Ana B. Cejudo, Mark A. McDaniel, M. Teresa Bajo

**Affiliations:** 1 Department of Experimental Psychology–Research Center for Mind, Brain and Behavior, University of Granada, Granada, Spain; 2 Department of Psychological and Brain Sciences, Washington University, St. Louis, Missouri, United States of America; University College London, UNITED KINGDOM

## Abstract

Prospective memory (PM), the ability to remember an intention in the future, is essential to children’s everyday lives. We explored age differences (6- to 7- vs. 10- to 11-year-olds) in PM depending on the nature of the task and the children’s motivation. Children performed event-based PM tasks (in which the cue was presented during the ongoing activity) and activity-based PM tasks (in which the cue consisted of finishing the ongoing activity). Additionally, the children were assigned to either a reward condition or a no-reward condition. The results showed better performance in event than in activity based tasks, with older children outperforming younger children in both. There was a marginal effect of reward for PM accuracy. These patterns suggest that the cue detection process and children’s motivation play a role in PM performance during development.

## Introduction

Prospective memory (PM) is the ability to remember a delayed intention. Forgetting to complete intentions could affect school-age children’s academic performance (e.g., forgetting to bring their homework to school) and social relationships (e.g., forgetting to give back a friend’s book). In PM, delayed intentions have to be remembered in response to particular contextual situations [1]. In event-based PM tasks, retrieval of an intention requires a trigger of an associated memory from some external cue (e.g., remembering to buy bread when passing the grocery store on the way home). In contrast, in time-based PM tasks, the person intends to perform a task at a specific time, within a specific time period or when a period of time has elapsed (e.g., remembering to buy bread before 8 p.m., when the grocery store closes). Finally, activity-based tasks require that intentions be retrieved and executed upon completing other tasks (e.g., remembering to buy bread after buying vegetables from the fruit stall). Successful completion of a PM task requires remembering an intention (e.g., press a key when red words appear on a screen) while performing another ongoing task (OT; e.g., answering general knowledge questions). In addition, at the appropriate moment or when the prospective cue appears, the person must stop performing the OT to instead perform the intention [2,3].

Several studies have shown developmental increments in PM performance using event-based PM tasks (see [4–6]). Age differences in PM tasks appear when cues are not part of the OT (non-focal cues), but not when cues are part of the OT (focal cues). Focality effects in event-based tasks have been studied with children of different ages [7]; however, very few developmental studies have focused on activity-based tasks [8,9], even though results with adult participants indicate that these are more difficult to remember than event-based tasks [10]. For instance, with adult participants Brewer et al. [10] reported differences in PM performance for event-based versus activity-based PM tasks. When the PM cue was event-based (e.g., saying “now” when the OT involved numbers), participants correctly responded 60% of the time, whereas their performance dropped to 23% when the PM cue was activity- based (e.g., saying “now” when the activity involving numbers came to an end). Similarly, the few studies conducted with children using activity-based PM tasks have shown lower performance in activity than in even based tasks, with age differences being more evident in activity than event based tasks. For example, Wash et al. [8] did not find age differences in pre-schoolers when they completed an event-based task in which children were asked to catch the explicit visual cue “Elmo” when its image appeared on the screen while they were playing a computer game. By contrast, the probabilities of asking for a sticker after finishing the game were quite low for 3-and 4- compare to 5-year-old children. However, activity based performance can improve if children are motivated [9]. Thus, in a study focusing on motivation and agency, Causey et al. [8] found that retrieval of the intention was higher in a high motivation condition where pre-schoolers were asked to remember to take a sticker when finishing the task relative to a low motivation condition, in which children were asked to remember to change the sign on the door. Therefore, initial findings suggest that activity-based and event-based PM differences seem to be relevant during development, and motivation seems to be a modulating factor.

In general, motivation seems to affect the probability that children remember the PM intention [11–14]. For example, Han et al. [12] required children to correctly place coloured balls (previously associated with a specific animal) below the corresponding animals. In addition, they had to give a model bone to a dog (PM task) whenever they encountered the target dog in the OT. In their Experiment 3, Han et al. [12] manipulated the difficulty of the OT by including three or five possible animal–colour pairs. They also manipulated the children’s motivation by offering a prize to half of the children if they performed well on the OT. The results showed that the difficulty of the OT affected the PM performance only when 3-, 4- and 5-year-old children were highly motivated to complete the task. In addition, in a naturalistic study in which parents described their children’s (7–8 and 10-11-year-olds) performance in everyday PM tasks, Penningroth, Bartsch and Mcmahan [11] reported better PM performance in the tasks the children considered more important. Further, older children outperformed younger children when the task was less motivating; however, these age differences disappeared for tasks that parents evaluated as highly important for their children. Hence, these studies suggest that children's motivation might modulate PM performance by elevating performance when children are highly motivated, and reducing the effects of age, and type of tasks.

Finally, other factors that seem to affect PM performance are the difficulty and nature of the OT task. In particular, there is some evidence that the difficulty and nature of the OT might modulate some PM effects. For example, Rendell, McDaniel, Forbes and Einstein [15] found differences in PM performance between younger and older adults in a non-focal event task (Experiment 1), but found that these age differences disappeared when the OT was less complex (Experiment 2). Similarly, some observed interactions between younger ages and focality in event-based PM tasks [7] have been interpreted as being due to the greater difficulty of non-focal tasks for younger children, since the OT is usually not adapted to children’s ages (e.g.[16,17]). The nature of the task (artificial vs. natural) might also explain some of the contradictory results. For example, Kliegel et al. [7] found no age differences in a focal event-based condition consisting of a driving computer game (artificial setting), while Krasny-Pacini, Servant, Alzieu and Chevignard [18] reported age differences in 8- to 11-year-old children who performed a focal event task in a more natural environment (in which the OT consisted of baking a chocolate cake). Therefore, the nature of the task might also be an important factor to consider when exploring PM effects. However, very few studies have explored natural tasks [8,18], and their use has not been extended to the types of prospective situations that children usually confront during school (e.g., writing their name on a test or asking for a letter for their parents).

In sum, because previous studies have shown that motivation (e.g. [9,12,13]) and type of PM task (activity- vs. event-based) influence children’s performance in PM tasks [8], we sought to explore whether motivation and type of PM task influenced the prospective memory performance of school-age children and whether these variables interacted with each other and modulated their possible effects. Because the nature of the OT task seems to also influence PM performance, an important feature of our study was that we explored children’s PM performance in the context of natural tasks that were close to the types of activities that children usually perform at school and by adapting the difficulty of the OT tasks to the participants’ ages. Thus, in our study children were evaluated using school-related OT activities (e.g., working with puzzles, completing math problems, reading, finding differences between pictures, etc.) that they performed in their school context. Because we were interested in developmental effects, we assessed the 6–7 and 10-11-year-olds with the adjusted event- and activity-based PM tasks. In addition, half of our participants were included in a reward (higher motivation) group, while half were assigned to a no-reward (lower motivation) group. Overall, we expected better performance in the event-based PM task than in the activity-based PM task. However, we anticipated that these PM-task differences would be more evident in the lower-motivation condition. Importantly, we expected that age differences would be larger in the more demanding activity-based conditions than the event-based conditions. These predictions were based on the assumption that more demanding PM conditions should produce larger developmental effects, and that motivation might modulate the effects of the type of PM task.

## Method

### Participants

A total of 115 children were recruited from a local primary school in Granada, Spain. The younger group consisted of 63 children (35 boys and 28 girls) aged 6 to 7 years (*M* = 6.89, *SD* = 0.38), and the older group consisted of 52 children (27 boys and 25 girls) aged 10 to 11 years (*M* = 10.99, *SD* = 0.39). Sample size was decided in advance based on the number of participants in previous studies examining children in which motivation was manipulated for the PM tasks [13,14]. The Ethics Committee of the University of Granada approved the procedure. The caregivers of all the children were provided information on the study and gave written informed consent in accordance with the Declaration of Helsinki.

### Materials and procedure

Our study assessed two groups of participants: 6- to 7-year-olds and 10- to 11-year-olds. Testing included individual sessions conducted in a quiet room at the school. Half of the participants (32 participants of the 6- to 7-year-old group and 24 of the 10-year-old group) were randomly assigned to the reward condition, while the other half were assigned to the no reward condition. The session lasted 20 minutes and included event and activity PM tests.

Both groups (reward and no reward) received the same initial instructions: “We are going to play some games in which you have to remember to do things. You should try to perform the tasks and to remember all the things I ask you to do, but you do not have to try to do them quickly. The time is not as important as your performance. Before starting each game, I will explain to you what you have to do and what you have to remember. When you finish one game, I will give you the instructions for the next game.

After receiving the initial instructions, children in the reward group were told that they were going to receive points for good performance and that they would be able to exchange their points for a reward at the end of the session: “For each task you perform right and for remembering the things I am going to ask you to remember, I will give you points. There are a total of four points. So, at the end of the game, if you have three or more points, I will give you a larger present [showing the children the larger of two bags], and if you have two or fewer points, I will give you the smaller present [showing the children the smaller bag]”. After these instructions were delivered, the bags were hidden to avoid distracting the children. In the instructions, we emphasized that accuracy in both PM and OT tasks was equally important.

PM was assessed in all children using four PM tasks. Each task had two versions: an event version and an activity version. Each version of the task included the same OT, but children were asked to remember different prospective intentions depending on whether they completed the event or the activity version of the task. In total, therefore, children were asked to remember a total of four intentions: two for the event versions of the tasks and the other two for activity versions of the tasks. Thus, PM performance could range from 0–2 for each type of task (event and activity-based; for a similar approach (see [18]). Every child performed 2 event- based tasks (e.g. one was the event version of “the puzzle” and the other was the event version of “math problems”) and two activity-based tasks (e.g., one was the activity version of the “reading task” and the other was the activity version of “the find the differences task”). The order of presentation of the OT tasks was kept constant (puzzle, reading, find the differences and math problems tasks), but all possible sequences of event-based and activity-based versions of the OT tasks were used and counterbalanced across participants. Hence there were 6 possible counterbalanced implementations that reflected the 6 sequences of event-based and activity based versions, and children were randomly assigned to one particular implementation (sequence). The OTs consisted of doing a puzzle, reading, finding differences between pictures and doing math problems. From the results of a pilot study involving twelve 6–7 and 10-11-year-old children, the OTs for all activities were adapted for the younger and older groups so that they had the same level of performance (i.e, for the older groups the OTs included a puzzle with more pieces, more complex sentences and math problems and more complex pictures, for which finding the differences was more difficult). The prospective tasks were the same for both age groups. Because all the children were able to complete all the OTs, these tasks were only evaluated looking at the speed with which the children completed the tasks.

In the event version of the *puzzle task*, the children were asked to complete a puzzle and remember to put all the pieces back inside the box, except for the two pieces that they did not have to use. These instructions were given as follows: “The last children who did the puzzle included pieces from another puzzle. Could you put all the pieces back inside the box, except for the pieces that will not be used?” Hence, the children could see the other-puzzle pieces (event cues) while putting the right pieces in the box, and they had to remember to leave the odd pieces out of the box. In the activity version, the children were asked to remind the experimenter to write the time down on the paper when they finished the puzzle: “I would like to know what time it is when you finish the first game. Could you remind me to write the time down on the paper before I explain to you the instructions for the new game?” To adapt the task difficulty to each age group, younger children did a 25-piece puzzle, and the older children did a 50-piece puzzle.

During the *reading task*, the children read sentences from a 15-page notebook. Each page included three sentences. To ensure they paid attention while they were reading, the children were asked to underline the words referring to animals. Additionally, in the event version, they were asked to circle the words referring to numbers. These instructions were given as follows: “Some of the sentences are too long. I would like to shorten them. Could you please help me and circle the words referring to numbers so that, later, I can change these words to digits?” To make it similar to the activity-based task only one PM cue appeared during the reading task. In the activity version, the children were asked to write their names on the right corner of the first page of the test once they finished the practice trials (the first three pages of the notebook, with one sentence per page): “We are now going to practice the task. Once you finish practicing, please do not forget to write your name in the right corner of the first page of the actual task. I need you to write your name there at the beginning of the test because the practice is the same for all children, and I need to know which test is yours”. To adapt the difficulty of the OT, the sentences presented to the older group were longer and more complex than the sentences presented to the younger group.

After the reading activity, children were given a task in which they had to *find the differences* between two pictures. In the event version of the task, they were asked to indicate the last difference they found with an arrow: “I would like you to find the differences between the two pictures; however, sometimes, one of the differences is very difficult to find. I would like to know which one was the most difficult for you. Would you please tell me what difference did you find the most difficult? Please mark it with an arrow”. In the activity version, the children were asked to remember to put the page used for the task inside an envelope, as follows: “I don’t want to lose your picture’s page. Could you put it inside this envelope when you finish the task?” The older group was presented with more complex pictures than the younger group.

Finally, the children completed *math problems* from a 10-page notebook with four arithmetic operations per page. We included two practice pages with two math problems per page. After practicing, the children were given the intention instruction. In the event version, they were asked to remember to circle the number three any time it appeared in operations (it appeared only once), as follows: “There is a math problem which is more difficult than the rest, and this problem contains a three. Could you find this problem for me so that I can eliminate it from the game?”. In the activity version, children were told to remember to ask for a letter for their parents after they had finished the practice part of the task, as follows: “I have a letter for your parents [showing the children the letter] that says we have finished the experimental session. Could you ask me for the letter after you finish practicing?” For the children in the reward group, the instructions changed to: “I have a letter for your parents saying that you have done a good job and that you finished the experimental session. Could you ask me for the letter after you finish practicing?” In order to adapt the task difficulty, the 10-11-year-old children were given math problems with three-digit numbers and the younger children were given math problems with one-digit numbers. For all the PM tasks children scored a point if they remembered the PM intention before starting the new game; in the last task children got a point if they remember to complete the intention (ask for the letter) before leaving the class.

Before starting each activity and after all the activities were completed, each child was required to repeat the task instructions. The idea was to check whether the children were able to remember what they were required to do in each task. If they could not remember the instructions for a particular task, the instructions were repeated. All children were able to remember the instructions after repetition.

## Results

To test that the OT difficulty was successfully adjusted to each age group and to explore whether the type of cue (event vs. activity) or motivation influenced the OT completion times (measured in seconds), a 2 (type of task: event vs. activity) x 2 (motivation: reward vs no reward) x 2 (age: 6–7 vs. 10–11) mixed ANOVA was performed on the completion times for the OT. The results of the analysis showed a significant effect of motivation (Table 1), *F* (1, 111) = 7.08, *MSe =* 15473.51, *p*< 0.01, η_p_^2^ = 0.06, indicating that the children in the reward group performed faster (*M* = 384.82, *SD* = 11.88) than the children in the no reward group (*M* = 428.75, *SD* = 11.47). All other effects and interactions were not significant: type of task x motivation, *F* (1, 111) = 1.14, *p* = 0.29, age, type of task x age, age x motivation, and type of task x age x motivation (Fs< 1). These results show that, although children in the reward group performed the OT faster than children in the no reward group, the type of cue did not influence OT completion-time performance. More relevant, no age differences were found in the completion times for OT performance, indicating that the OT was properly adapted to each age.

**Table 1:** OT completion time.

		Event-based	Activity-based
6 to 7 years	No Reward	447 (114)	421 (130)
	Reward	362 (84)	379 (116)
10 to 11 years	No Reward	441 (132)	405 (126)
	Reward	403 (154)	394 (131)

*Note*: Mean total OT completion time (in seconds) as a function of age, type of task and reward conditions (standard deviations in parentheses).

### PM performance

PM scores were analysed by comparing event and activity conditions between the reward and no reward groups and the two age groups (6–7 and 10-11-year-olds). PM correct responses for each condition were calculated and submitted to a 2 (type of task: event vs. activity) x 2 (motivation: reward vs no reward) x 2 (age: 6–7 vs. 10–11) mixed ANOVA. The results of this analysis revealed a statistically significant effect of type of PM task (Table 2), *F* (1, 111) = 18.08, *MSe =* 0.11, *p* < 0.01, η_p_^2^ = 0.14, 1-β = .98 with better performance in the event PM (*M* = 0.67, *SD* = 0.35) than in the activity PM (*M* = 0.48, *SD* = 0.38) condition. The effect of age was also reliable: The older group was more accurate than the younger one (*M* = 0.69, *SD* = 0.31 vs. *M* = 0.48, *SD* = 0.37), *F* (1, 111) = 18.77, *MSe =* 0.13, *p* < 0.01, η_p_^2^ = 0.14, 1-β = .99. The motivation effect was very close to statistical significance, *F* (1, 111) = 3.78, *MSe =* 0.13, *p* = 0.054, η_p_^2^ = 0.03, 1-β = .49, such that higher motivation (reward) tended to produce better performance than lower motivation (no reward). None of the interactions was significant: type of task by age, *F* (1, 111) = 2.00, *p* = 0.16, 1-β = .29; type of task by motivation, *F* (1, 111) = 1.26, *p* = 0.26, 1-β = .20; age by motivation, *F* (1, 111) = 0.53, *p* = 0.47, 1-β = .11; type of cue x age x motivation, *F* (1, 111) = 0.14, *p* = 0.71, 1-β = .07. Because the distribution of PM scores was not normal, we performed supplementary non-parametric analyses (see [14] for a similar approach). A Kruskal–Wallis ANOVA confirmed that the main effects of type of PM task H(1) = 14.00, *p* < 0.01 and age H(1) = 16.47, *p* < 0.01 were significant.

**Table 2:** Prospective memory performance.

		Event-based	Activity-based	Mean
6 to 7 years	No Reward	0.50(0.36)	0.34(0.35)	0.34(0.35)
	Reward	0.59(0.39)	0.50(0.38)	0.50(0.38)
10 to 11 years	No Reward	0.82(0.24)	0.50(0.38)	0.50(0.38)
	Reward	0.81(0.25)	0.62(0.38)	0.62(0.38)
	Mean	0.68(0.31)	0.49(0.37)	0.49(0.37)

*Note*: Means and standard deviations of the proportions of correct response as a function of age, type of task and reward conditions.

Although motivation did not interact with the other variables, because previous studies have shown that age differences in PM performance decrease with motivation, we compared children´s performance in both motivation groups (reward and non-reward, see last column in Table 2). The results indicated significant differences between the younger (*M* = 0.42, *SD* = 0.36) and older (*M* = 0.66, *SD* = 0.31) groups in the no-reward condition (*t* (116) = 3.63, *p <*0.01, d = 0.71) and also in the reward condition (*t* (110) = 2. 55, *p <*0.05, d = 0.51; *M* = 0.54, *SD* = 0.38 and *M* = 0.72, *SD* = 0.32), although the effect size was larger in the no-reward condition. In addition, because we were expecting motivation to have larger effects in the activity-based than in the event-based condition, we conducted post-hoc analyses to examine the effects of motivation on each type of cue condition. These analyses revealed no differences between the reward (*M* = 0.70, *SD* = 0.32) and no reward (*M* = 0.66, *SD* = 0.30) conditions when the cue was event-based (*t* (113) = 0.53, *p =* 0.05, d = 0.13), whereas motivation had a significant benefit (*t* (113) = 1.97, *p =* 0.05, d = 0.37) when the cue was activity-based (*M* = 0.56, *SD* = 0.38; *M* = 0.42, *SD* = 0.38).

## Discussion

We explored whether age differences in remembering an intention were modulated by the type of PM task (activity- vs. event-based) and by children’s motivation. The idea was that children would perform event-based PM tasks better than activity-based tasks in line with previous results with pre-school children and adults [8,10]. We also expected that motivation would increase performance and that age differences would be more evident in more difficult conditions (activity-based tasks) and when the children were not motivated. The results partially supported our expectations.

First, performance was higher for event-based than activity-based tasks, indicating that activity-based intentions are more difficult to retrieve at the appropriate moment than are event-based tasks. This effect has been attributed to the high salience of the event-based cues since these event-based tasks provide more explicit visual cues than the activity-based tasks [8]. In line with this interpretation, Brewer et al. [10] showed that when activity-cues were made salient by asking participants to underline the last generated word (OT) and write the number of words generated, participants improved their performance in the activity based tasks. Therefore, our results are consistent with previous findings indicating that the salience of the cue (represented by event vs activity based tasks) is an important factor determining PM performance [7]. The present result was obtained when the OT task difficulty was adjusted to the children’s ages. This is important because it suggests that the type of cue effect (event-based vs activity-based) and the developmental differences cannot be attributed to the nature of the OT task—specifically, differences in difficulty of the OT. In the present paradigm the equivalence of OT-task difficulty across age groups is supported by the finding that children in both age groups were able to perform the OT tasks in similar time frames. Also of importance, the OTs and the context in which they were performed were chosen to be similar to the activities and contexts the children usually experienced at school or at home. To our knowledge, this is the first study directly comparing activity- and event-based tasks that adjusts the OT to the children’s age and uses natural tasks and school contexts.

Intriguingly, the performance of the older children was higher than that of the younger children, independent of the type of PM task and motivation. Age effects are easily explained by the fact that, within the studied age range, attentional processes and executive functions are still developing (e.g. [19]), and PM performance during childhood is likely to correlate with the development of executive functions [20,21]. However, we expected smaller or even null effects in the easier event-based condition, since event-based tasks are assumed to involve more spontaneous, less demanding processes than non-focal or activity-based tasks [22]. Previous studies have shown contradictory results, with some studies showing age differences in focal event tasks [8,18] and others showing no differences [7]. These contrasting results could be explained by differences in the tasks used. For example, Kliegel et al.[7] used a computer game as the OT, while Walsh et al. and Krasny-Pacini et al.[8,18] used more natural tasks, such as purchasing items while shopping. It is likely that computer games—in which children focus only on the computer screen—are more absorbing (i.e., more focal) than natural tasks, in which the children move more freely and information is more dispersed. However, this needs to be tested, since the effects of different type of cues on children have rarely been investigated.

We also sought to test the role of motivation in the performance of activity- and event-based tasks, as several studies have found motivation to have positive effects on PM performance during childhood (e.g. [9,11–13]). Our hypothesis was that, if children were motivated, their PM performance would increase and age differences would decrease [11]. However, our results show only marginally significant motivation effects. The fact that our motivation effects were smaller than those obtained in previous studies might be due to several factors. First, the procedures were very different. For instance, Penningroth et al. [11] based their results on parents’ recordings of their children’s activities, whereas, our study was based directly on children’s performance on PM tasks. Second, it is possible that the children were not differentially motivated by our motivation conditions. Although a previous study with adults has shown that participants’ pro-social PM performance is improved when they obtain a large reward (e.g., 20 euro) relative to a small reward (e.g., 1 euro) or no reward, in our study the children might have perceived neither reward to be sufficiently stimulating because the reward was presented inside a bag (big or small) and was unknown [23]. Despite these factors, the reward instructions induced faster responses in the OT and showed a tendency to increase PM performance in the activity-based task, but not in the event-based task. Note that it is possible, that children under the faster OT times in the reward condition might have produced lower performance in the PM task (speed/accuracy trade-off) so that if they had remained at the slower pace than children in the non-reward condition, the differences in PM performance might have been larger. In any case, it is clear that motivation had an effect on OT performance by reducing response times and that it seemed to increase PM performance in activity based tasks. This pattern of results would suggest that motivation might only benefit the effortful monitoring processes required to perform PM tasks when less salient PM cues are involved (activity-based tasks) and not the performance of tasks involving explicit visual cues (event-based PM tasks), in which retrieval would be driven by cues with less involvement of attentional control [22]. Interestingly, our results also suggest that even younger children could strategically deploy these resources when motivated to improve their performance.

In conclusion, the results show that PM during child development is affected by PM task type. PM tasks in which cues appeared as part of the OT produced better performance than PM tasks in which the intention was signalled by the end of the OT and required more cognitive resources for detection. In addition, PM performance was better in 10-11year-olds than in 6-to 7-year-olds in both the event and activity conditions. This result is in line with the suggestion that cognitive resources (e.g., working memory, inhibition and task-switching) are related to good PM performance in children [20,21]. Finally, our motivation condition had a small effect that was more evident in the activity-based tasks.
